# Early on-treatment plasma interleukin-18 as a promising indicator for long-term virological response in patients with HIV-1 infection

**DOI:** 10.3389/fmed.2023.1170208

**Published:** 2023-06-13

**Authors:** Weiyin Lin, Liya Li, Pengle Guo, Yaozu He, Haolan He, Hong Li, Huolin Zhong, Cong Liu, Peishan Du, Weiping Cai, Xiaoping Tang, Linghua Li

**Affiliations:** Guangzhou Eighth People’s Hospital, Guangzhou Medical University, Guangzhou, China

**Keywords:** HIV-1, IL-18, indicator, long-term, virological response

## Abstract

**Background and aims:**

It is necessary to identify simple biomarkers that can efficiently predict the efficacy of long-term antiretroviral therapy (ART) against human immunodeficiency virus (HIV), especially in underdeveloped countries. We characterized the dynamic changes in plasma interleukin-18 (IL-18) and assessed its performance as a predictor of long-term virological response.

**Methods:**

This was a retrospective cohort study of HIV-1-infected patients enrolled in a randomized controlled trial with a follow-up of 144  weeks of ART. Enzyme-linked immunosorbent assay was performed to evaluate plasma IL-18. Long-term virological response was defined as HIV-1 RNA <20 copies/mL at week 144.

**Results:**

Among the 173 enrolled patients, the long-term virological response rate was 93.1%. Patients with a long-term virological response had significantly lower levels of week 24 IL-18 than non-responders. We defined 64  pg./mL, with a maximum sum of sensitivity and specificity, as the optimal cutoff value of week 24 IL-18 level to predict long-term virological response. After adjusting for age, gender, baseline CD4+ T-cell count, baseline CD4/CD8 ratio, baseline HIV-1 RNA level, HIV-1 genotype and treatment strategy, we found that lower week 24 IL-18 level (≤64 vs. >64 pg./mL, a OR 19.10, 95% CI: 2.36–154.80) was the only independent predictor of long-term virological response.

**Conclusion:**

Early on-treatment plasma IL-18 could act as a promising indicator for long-term virological response in patients with HIV-1 infection. Chronic immune activation and inflammation may represent a potential mechanism; further validation is necessary.

## 1. Introduction

Human immunodeficiency virus (HIV) infection continues to be a major global public health issue, having resulted in 40.1 million deaths to date; in 2021, 650,000 people died from HIV-related causes, and 1.5 million people acquired HIV. Maintaining viral suppression through effective antiretroviral therapy (ART) is critical for reducing morbidity and mortality in this population. However, obstacles to fully controlling HIV include patient compliance, drug resistance and drug–drug interactions. In addition, many HIV services, such as testing and monitoring, have been disrupted during the coronavirus disease 2019 epidemic. Therefore, more efforts are needed to identify useful predictors of treatment efficacy. Identifying these predictors can help to optimize ART in real-world clinical practices, thereby contributing to reaching the new proposed global 95-95-95 targets set by the Joint United Nations Program on HIV and AIDS (UNAIDS).

Previous studies have shown that specific biomarkers of inflammation are associated with increased mortality and morbidity, including clinical treatment failure, incident active tuberculosis and other longer-term outcomes, such as cardiovascular disease, in HIV-infected patients (1–5). Although these studies demonstrate the potential applications of biomarkers for inflammation, a simple biomarker that can efficiently predict the efficacy of long-term ART remains an unmet medical need, especially in areas with limited resources. Interleukin 18 (IL-18), a member of the IL-1 family of cytokines, is produced by both hematopoietic and non-hematopoietic cells, including monocytes, macrophages, keratinocytes and mesenchymal cell (6). IL-18 is a potent pro-inflammatory cytokine which involved in host defense against infections and regulates the innate and acquired immune response (6). Recently, we conducted a short-term retrospective cohort study (48 weeks) consisting of HIV-1-infected patients who received lopinavir/ritonavir (LPV/r) plus lamivudine (3TC) treatment or tenofovir disoproxil fumarate (TDF) plus 3TC plus efavirenz (EFV) treatment. We found that the levels of plasma IL-18 showed U-shaped regression curves while the virus decreased and immune function recovered after initiating ART and that the viral suppression rate at week 12 of treatment was higher in patients with lower baseline IL-18. These findings suggested that there may be an inherent institutional association and that IL-18 may be associated with the efficacy of ART (7).

In this study, we aimed to further characterize the dynamic changes in IL-18 for up to 144 weeks and to assess the performance of IL-18 as a predictor of long-term virological response.

## 2. Patients and methods

### 2.1. Patients

This was a retrospective cohort study consisting of HIV-infected patients enrolled in a randomized, controlled, open-label, noninferiority trial (ALTERLL study) (8, 9). The inclusion criteria were as follows: infected with HIV-1, older than 18 years, naive to ART, had a CD4+ cell count over 200/μL at the time of screening, and completed the ALTERLL study. The exclusion criteria were as follows: lack of sufficient samples to detect IL-18, pregnant or breastfeeding, coinfection with hepatitis B virus (HBV) or hepatitis C virus (HCV), or chronic liver disease or acquired immune deficiency syndrome (AIDS)-associated opportunistic diseases within 30 days before screening (8, 9). The allocation and treatment strategy are shown in Supplementary Figure S1.

This study was approved by the Ethics Committee of Guangzhou Eighth People’s Hospital (Approval No. 20142154) and registered with the Chinese Clinical Trial Registry, number ChiCTR1900024611. Written informed consent was obtained from all patients.

### 2.2. Clinical and laboratory evaluation

Clinical and laboratory assessments were performed at baseline and at 12 weeks, 24 weeks, 48 weeks, 96 weeks, and 144 weeks of treatment. Absolute CD4+ T lymphocyte (*CD3 + CD4 + CD8−*) count and CD8+ T lymphocyte (*CD3 + CD4 − CD8+*) count were measured using a BD CantoII flow cytometer with CD3/CD4/CD8 trichrome fluorescence reagent from BD according to the instructions. BD Multitest™ CD3/CD8/CD45/CD4 IMK Kit used in this study contains FITC-labeled CD3, clone SK7; PE-labeled CD8, clone SK1; PerCP-labeled CD45, clone 2D1 (HLe-1); and APC-labeled CD4, clone SK3. Plasma HIV-1 RNA load was quantified using the Roche COBAS-TaqMan Assay (HIV-1 Test, version 2.0, Indianapolis, IN, United States), and the detection limit was 20 copies/mL. Long-term virological response was defined as HIV-1 RNA <20 copies/mL at week 144 of treatment. Subgenotypes of the HIV-1 virus were tested with the standard strains selected from the Los Alamos HIV database using Bioedit and MEGA5 software, and mutation sites were identified using the Stanford HIV-resistant database (Supplementary Table S1), which has been described elsewhere (7).

### 2.3. Plasma IL-18 evaluation

For the current study, the enzyme-linked immunosorbent assay method was performed to evaluate plasma IL-18 at baseline and at 12 weeks, 24 weeks, 48 weeks, 96 weeks and 144 weeks of treatment using the IL-18 kit from MBI, Japan, with a concentration unit of pg./mL. Plasma samples were diluted 1:5 based on titration assays to fit within the standard curve range. Measurement and reading of cytokine levels were carried out strictly according to the instructions by laboratory technicians blinded to the outcome of study participants.

### 2.4. Statistical analysis

Data are expressed as counts and percentages for categorical variables and as medians and interquartile ranges (IQRs) for continuous variables. Qualitative and quantitative differences were analyzed using *χ*^2^ test or Fisher’s exact tests for categorical parameters and Student’s *t* test or Mann–Whitney test for continuous parameters, as appropriate. The longitudinal trend of IL-18 was tested by polynomial contrasts of one-way ANOVA. For analyses of the performance of longitudinal IL-18 levels and changes at specific timepoints in predicting long-term virological response, the areas under the receiver operator characteristic curve (AUROC) of two parameters were calculated. The optimal cutoff value of plasma IL-18 was identified using the Youden index. Logistic regression analysis was used to determine predictors of long-term virological response. All statistical tests were 2-sided. Statistical significance was taken as *p* < 0.05. All analyses were performed with SPSS software, version 26.0 (IBM, Armonk, NY).

## 3. Results

### 3.1. Patient characteristics

A total of 173 patients were enrolled in this study. The demographic, virological and clinical characteristics of the patients are shown in Table 1. Most of the patients were young males with HIV-1 RNA between 3–5 log_10_ copies/mL; approximately half of the patients had CD4+ T-cell counts less than 350/μL; and 30.1 and 44.5% of the patients were infected with HIV-1 genotypes CRF01_AE and CRF07_BC, respectively. The rates of viral suppression (HIV-1 RNA <20 copies/mL) reached 92.5% at week 48 and were maintained at 93.1% until week 144 of ART. Additionally, the viral load among patients with detectable HIV-1 RNA decreased from 4.4 to 1.6 log_10_ copies/mL.

**Table 1:** Clinical characteristics of the studied patients.

**Characteristic**	*N* = 173
**Age at enrollment**, years	29.0 (25.0–35.5)
**Gender**, *n* (%)	
Male, *n* (%)	160 (92.5)
Female, *n* (%)	13 (7.5)
**Baseline HIV-1 RNA**, log_10_ copies/mL	4.4 (3.8–4.7)
<3 log_10_ copies/mL, *n* (%)	7 (4.0)
3–5 log_10_ copies/mL, *n* (%)	143 (82.7)
>5 log_10_ copies/mL, *n* (%)	23 (13.3)
**Baseline CD4+ T-cell count**, cells/mm^3^	328.0 (264.0–389.5)
≤350 cells/mm^3^, *n* (%)	100 (57.8)
351–500 cells/mm^3^, *n* (%)	62 (35.8)
>500 cells/mm^3^, *n* (%)	11 (6.4)
**Baseline CD4/CD8 ratio**	0.32 (0.23–0.44)
**HIV-1 genotype**	
CRF01_AE, *n* (%)	52 (30.1)
CRF07_BC, *n* (%)	77 (44.5)
Others, *n* (%)	44 (25.4)
**Antiretroviral therapy**	
LPV/r plus 3TC, *n* (%)	88 (50.9)
TDF plus 3TC plus EFV, *n* (%)	85 (49.1)

Data are expressed as counts and percentages for categorical variables and as the median and interquartile range for continuous variables. HIV, human immunodeficiency virus; LPV/r, lopinavir/ritonavir; 3TC, lamivudine; TDF, tenofovir disoproxil fumarate; EFV, efavirenz.

### 3.2. Longitudinal changes in plasma IL-18

**Figure 1:**
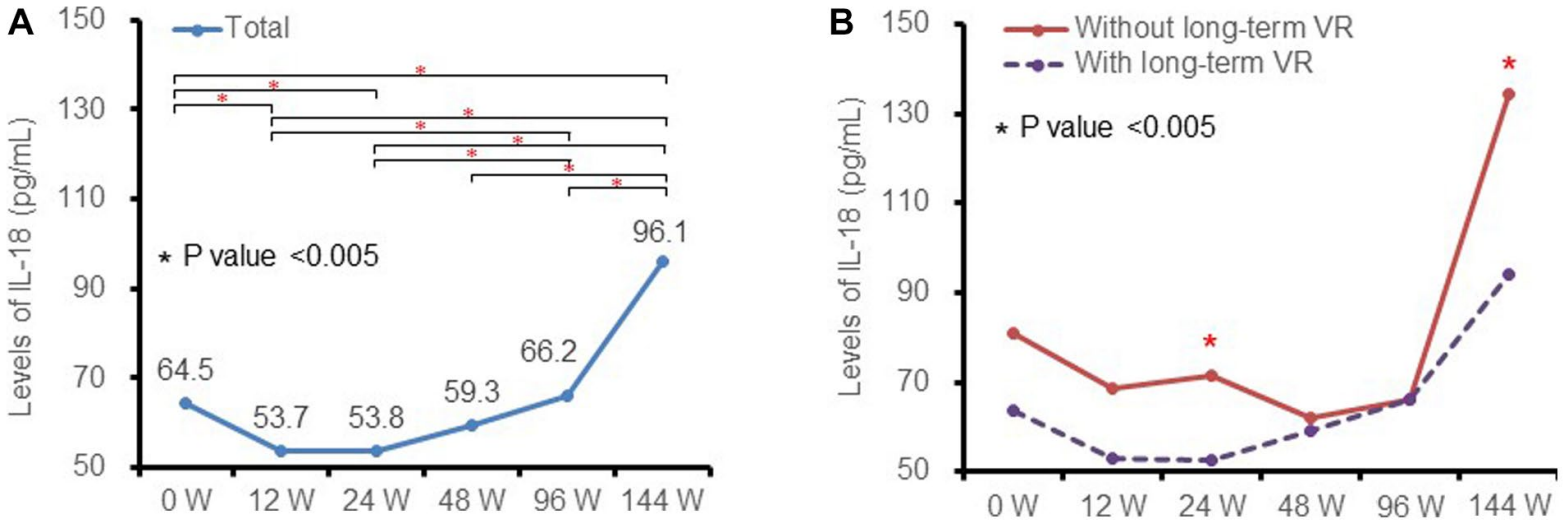
Kinetics of plasma IL-18 at different timepoints and subgroups. **(A)** in total patients and **(B)** in patients with (purple dotted line) and without (red solid line) long-term virological response. IL-18, interleukin-18; VR, virological response.

At baseline, the median level of plasma IL-18 was 65.4 (IQR 47.7–88.9) pg./mL. The levels of IL-18 decreased after initiating ART, reached a nadir at week 12 (53.7 [IQR 39.5–76.8] pg./mL) and week 24 (53.8 [IQR 40.0–70.5] pg./mL), and thereafter increased significantly to 96.1 (IQR 66.6–139.5) pg./mL at week 144 (Figure 1A). The dynamic changes in median plasma IL-18 levels fit a quadratic equation with a U-shape (*F* = 70.356, *p* value <0.001).

Levels of IL-18 among patients with and without long-term virological response were further analyzed as shown in Figure 1B. The dynamic changes in the above subgroups were similar to those of the total patient population. Patients with a long-term virological response had lower levels of IL-18 than non-responders, and the difference was statistically significant at week 24 and week 144 (all *p* values <0.05).

### 3.3. Performance of plasma IL-18 levels for predicting virological response

**Figure 2:**
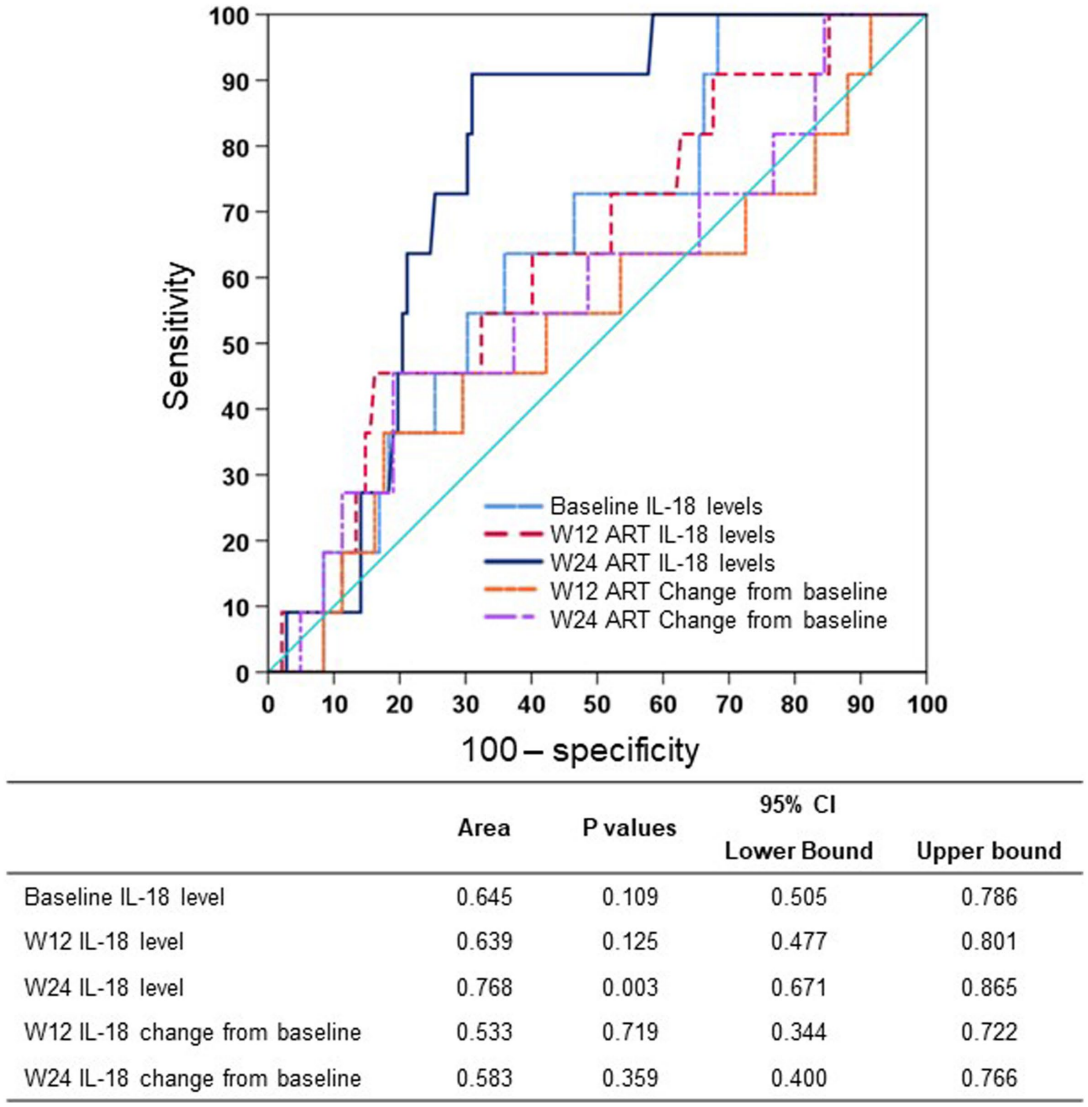
AUROCs of plasma IL-18 at different timepoints predicting long-term virological response. ART, antiretroviral therapy; AUROC, area under the receiver operator characteristic curve; CI, confidence interval; IL-18, interleukin-18; W12, week 12 of treatment; W24, week 24 of treatment.

We studied the early on-treatment (defined as initiation of ART within 24 weeks) plasma IL-18 levels and changes at week 12 and week 24 to evaluate the predictive performance for long-term virological response using receiver operating characteristic (ROC) curves. The AUROC of the IL-18 level was highest at week 24 (0.768, 95% CI: 0.671–0.865) and higher than the IL-18 change from baseline to week 12 or week 24 (Figure 2). By summing the sensitivity and specificity of the week 24 IL-18 level in predicting long-term virological response, the Youden index was highest when the cutoff value of the week 24 IL-18 level was 64.0 pg./mL, and the corresponding sensitivity and specificity were 90.9 and 68.2%, respectively (Supplementary Table S2). Therefore, we adopted 64.0 pg./mL as the optimal cutoff value of week 24 IL-18 level in the following analyses.

### 3.4. Correlation between baseline and early on-treatment characteristics and virological response

To further evaluate baseline and early on-treatment characteristics in predicting long-term virological response, a logistic regression analysis was conducted with the inclusion of age, gender, baseline CD4+ T-cell count, baseline CD4/CD8 ratio, baseline HIV-1 RNA level, HIV-1 genotypes, ART strategy and week 24 IL-18 level in the model (Table 2). In the univariate analysis, baseline log_10_ HIV-1 RNA level (odds ratio [OR] 0.26, 95% confidence interval [CI]: 0.09–0.76) and lower week 24 IL-18 level (≤64 vs. >64 pg./mL, OR 21.43, 95% CI: 2.67–172.11) were associated with virological response. In the multivariate analysis, lower week 24 IL-18 level (≤64 vs. >64 pg./mL, adjusted OR 19.10, 95% CI: 2.36–154.80) was the only independent predictor for long-term virological response.

**Table 2:** Baseline and early on-treatment variables associated with long-term virological response.

Variables	Virological response at week 144			
	Univariate		Multivariate	
	OR (95% CI)	*p*	aOR (95% CI)	*p*
**Age at enrollment** (years)	0.97 (0.91–1.03)	0.360		
**Gender** (Female vs. male)	0.89 (0.11–7.45)	0.911		
**Baseline CD4 categories** (≤350 vs. 351–500 vs. >500/μL)	3.71 (0.85–16.14)	0.081		
**Baseline CD4/CD8 ratio**	3.58 (0.06–214.27)	0.541		
**Baseline log**_**10**_**HIV-1 RNA level** (log_10_ copies/mL)	0.26 (0.09–0.76)	**0.014**	0.39 (0.12–1.23)	0.108
**HIV-1 genotype**				
CRF01_AE	Ref			
CRF07_BC	0.45 (0.08–2.43)	0.352		
Others	0.69 (0.13–3.69)	0.660		
**Antiretroviral therapy** (Lpv/r + 3TC vs. TDF + 3TC + EFV)	1.04 (0.32–3.35)	0.950		
**Week 24 IL-18 level** (≤64 vs. >64 pg./mL)	21.43 (2.67–172.11)	**0.004**	19.10 (2.36–154.80)	**0.006**

The variables enrolled in logistic regression analysis were age (years), sex (female vs. male), baseline CD4+ cell count (≤350 vs. 351–500 vs. >350/μl, as rank variables), baseline CD4/CD8 ratio, baseline log_10_ HIV-1 RNA level (log_10_ copies/ml), HIV-1 genotype (CRF01_AE vs. CRF07_BC vs. others), antiretroviral therapy (LPV/r plus 3TC vs. TDF plus 3TC plus EFV) and baseline IL-18 level (≤64 vs. >64 pg/mL). Backward stepwise (likelihood ratio) regression was performed. 3TC, lamivudine; aOR, adjusted odds ratio; CI, confidence interval; EFV, efavirenz; HIV, human immunodeficiency virus; LPV/r, lopinavir/ritonavir; TDF, tenofovir disoproxil fumarate. *P* values less than 0.05 are expressed in bold.

### 3.5. Rates of virological response among patients with favorable baseline or early on-treatment characteristics

**Figure 3:**
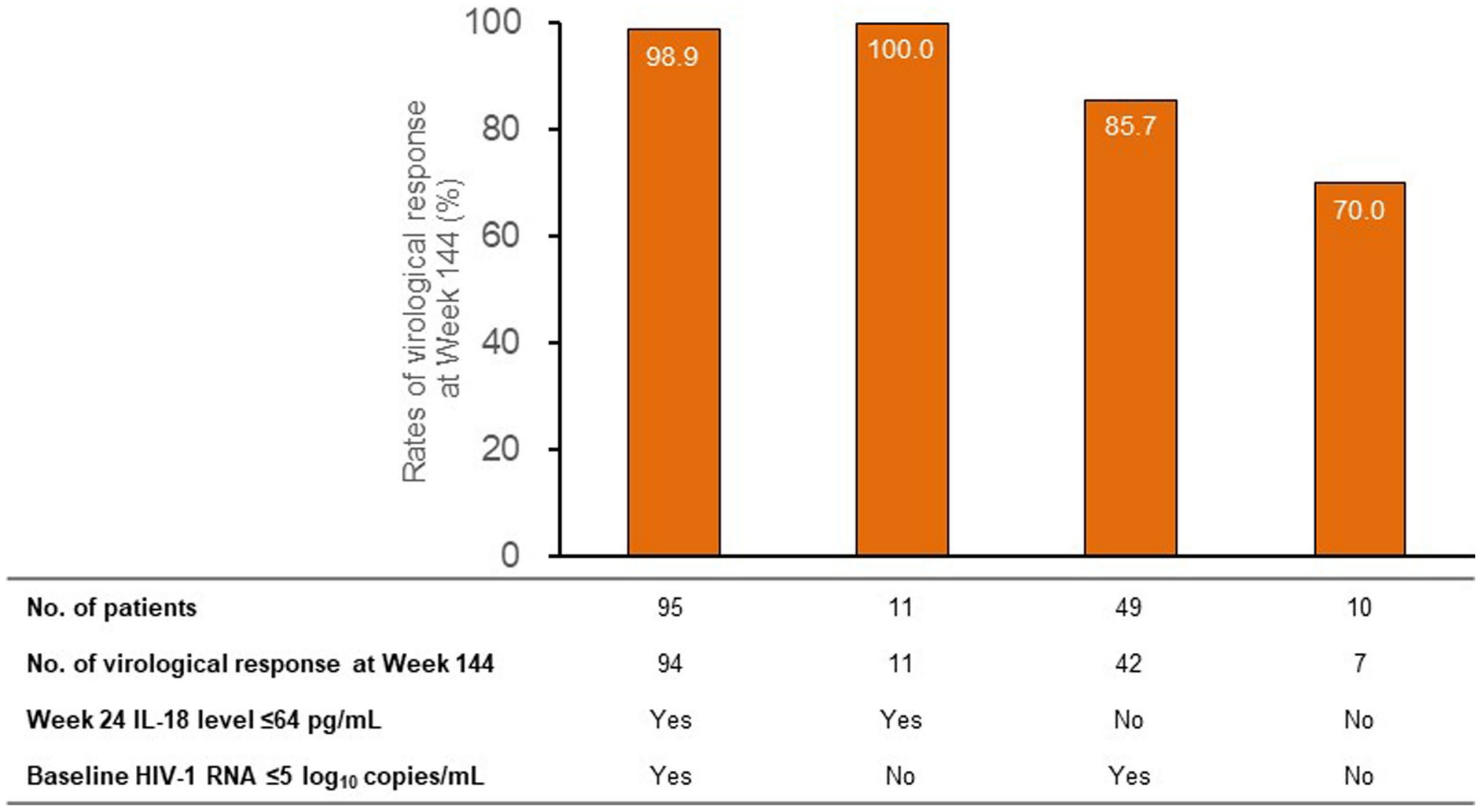
Long-term virological response among subgroups of patients stratified by baseline HIV-1 RNA and week 24 IL-18. HIV, human immunodeficiency virus; IL-18, interleukin-18.

Patients with long-term virological response had lower levels of baseline log_10_ HIV-1 RNA than non-responders (median 4.3 vs. 4.8 log_10_ copies/mL). Figure 3 shows the long-term virological response rates under the combination of baseline HIV-1 RNA (cutoff value as 5 log_10_ copies/mL) and week 24 IL-18 (cutoff value as 64 pg./mL). The rates of long-term virological response were high (98.9 and 100.0%) among patients with week 24 IL-18 ≤ 64 pg./mL and quite low (85.7 and 70.0%) among patients with week 24 IL-18 > 64 pg./mL, irrespective of baseline HIV-1 RNA levels.

## 4. Discussion

Numerous studies have revealed that chronic immune inflammation is a hallmark of HIV-1 infection that persists even after several years of successful ART and is associated with noninfectious complications of HIV-1, including cardiovascular disease, non-AIDS malignancies and frailty (10, 11). Our recent short-term study demonstrated that plasma IL-18 decreased after initiation of ART up to 24 weeks but rebounded by 48 weeks post-ART initiation (7). The current study further uncovered the long-term dynamic changes in IL-18, that is, it continued to rise after 48 weeks of ART. Consistent with our previous work, early virology response was slightly higher in patients with LPV/r + 3TC treatment than those with TDF + 3TC + EFV; not surprisingly, there was no difference between the long-term virological response of these two kinds of antiretroviral therapies (93.2% vs. 92.9%, *p* value = 0.950). In addition, the absolute levels of IL-18 at most visits and dynamic changes were similar in different treatment groups (data not shown). The patients with good viral control had significantly lower levels of week 24 IL-18, which is consistent with previous studies (5, 12), and supports persistent chronic immune activation and inflammation in HIV-1-infected patients with good viral control. We also noted there was no difference in IL-18 levels in week 48 and 96 between patients with and without long-term virological response (Figure 1B). One possible reason is that, due to the severe chronic immune activation and inflammation, the arrival time of nadir IL-18 in non-responders was later than that in responders. Therefore, the asynchronous nadir may be one of the reasons of this phenomenon.

Various markers of chronic immune activation and inflammation, including C-reactive protein, soluble leukocyte differentiation antigen 14, and cytokines such as IL-6, are associated with increased mortality (1, 2). In a nested case–control study, Balagopal et al. found that continued elevation of IL-18 after initiation of ART is associated with clinical failure, which was defined by incident World Health Organization Stage 3 or 4 event or death (5). In another case–control study conducting an exploratory factor analysis based on multiple biomarkers in HIV-infected individuals, Rupak et al. found that biomarkers of inflammation were associated with HIV clinical treatment failure (including incident active tuberculosis) or death within 96 weeks post-ART initiation (13). However, the above studies involved a large number of cytokines, which are difficult to realize in real-world clinical practice, especially in under-resourced countries. In addition, they lacked longitudinal observation and further exploration of the best predictive cutoff values of dominant cytokines. In contrast to previous studies, we focused on longitudinal plasma IL-18 levels and demonstrated the predictive value of week 24 IL-18 levels for the efficacy of long-term ART. Furthermore, the optimal cutoff value was determined with a maximum sum of sensitivity and specificity, which will be convenient for its application in clinical practice.

As expected, both baseline HIV-1 RNA and CD4+ cell count were associated with virological response in univariate analysis, which confirms previous studies in patients with HIV and clearly indicates that our study cohort has a limited issue of bias (14, 15). However, after adjusting for IL-18 level, neither baseline HIV-1 RNA nor CD4+ cell count was associated with virological response. Further analysis with classified variables or continuous variables showed similar results (data not shown). One possible explanation is that long-term virological response may be less affected by baseline viral load and baseline immune function than short-term (48 week) virological responses. This was supported by our previous work showing that lower baseline HIV-1 RNA levels could independently predict higher rates of virological responses at week 48 of ART (7). In addition, the predictive effectiveness of week 24 IL-18 on long-term virological response is extremely strong, and when they are simultaneously included in the multivariate model, the predictive effectiveness of baseline HIV-1 RNA and baseline CD4+ cell count may appear to be very weak, which is not uncommon in multivariate analysis. The long-term virological response rates were identified in subgroups according to the combination of stratified baseline HIV-1 RNA and week 24 IL-18 levels (15). Regardless of the baseline HIV-1 RNA level, the long-term virological response rate is high as long as IL-18 is less than the established cutoff value; in contrast, it is quite low as long as IL-18 is greater than the established cutoff value. These findings indicate that early on-treatment plasma IL-18 level alone could predict long-term virological response in patients with HIV-1 infection.

The mechanism underlying the predictive value of plasma IL-18 levels is not completely understood. IL-18 is a multifunctional cytokine secreted mainly by monocytes/macrophages that has various biological functions, such as inducing IFN-γ production by Th1 cells, enhancing NK cell activity, and enhancing FasL-mediated cytotoxic effects, and has an important role in the immune response process, anti-infection, inflammatory response and autoimmune diseases (16, 17). Previous studies have shown that IL-18 is involved in the pathogenic process of HIV to some extent (18). Choi et al. found that IL-18 plays an important role in viral clearance and inhibits HIV-1 replication in acutely infected cells *in vitro* (19). However, the metabolic pathways of macrophages determining the susceptibility to infection, the persistence of infected cells and the establishment of latency of HIV are unclear (11). We hypothesized that chronic immune activation and inflammation after initiation of ART would be an immunological manifestation of the latent HIV reservoir and would deplete the immune cells that are favorable to viral control. This hypothesis is supported by the close correlation between IL-18 and adverse outcomes in this population and recent studies regarding M2 macrophage polarization and the development of renal fibrosis (20–22). Further mechanistic investigations are needed.

Despite these interesting findings, there are limitations in this study. First, the sample size of this study was small, over 90% were male, all of the participants were from China, and the ART regiments did not contain current first-line integrase strand transfer inhibitors or fusion inhibitors. Therefore, the conclusions cannot be generalized to other populations, and independent external validation with a larger sample size and more diversified treatment options from other countries or regions are necessary. Second, due to its observational nature, this study cannot elucidate the mechanisms that link IL-18 and long-term virological response. Whether the change in IL-18 is a cause or consequence of viral control or confounded by some other related host-immunologic pathway remains to be confirmed. In addition, coinfection, adherence and other potential risk factors for unfavorable viral control were not analyzed in the current study because of the eligibility criteria and strict follow-up management. Nevertheless, this cohort study provides evidence for the dynamic changes and clinical significance of plasma IL-18 after long-term ART in the process of HIV-1 infection.

In conclusion, early on-treatment plasma IL-18 levels could act as a promising indicator for long-term virological response in patients with HIV-1 infection, which could be used for optimizing ART in real-world clinical practices. Further validation and mechanistic studies are needed.

## Data availability statement

The raw data supporting the conclusions of this article will be made available by the authors, without undue reservation.

## Ethics statement

The studies involving human participants were reviewed and approved by Guangzhou Eighth People’s Hospital, Guangzhou Medical University. The patients/participants provided their written informed consent to participate in this study.

## Author contributions

LingL, XT, WL, and LiL conceived the study and designed the protocol. HH, HL, and HZ gave instructions. PG, YH, LingL, and XT contributed to the statistical analysis and interpretation of the data. WL and LiL drafted the manuscript. CL, PD, and WC contributed to conducting the study and collecting data. All authors contributed to the article and approved the submitted version.

## Funding

This work was supported by grants from Guangzhou Basic Research Program on People’s Livelihood Science and Technology (grant number: 202002020005), National Natural Science Foundation of China (grant number: 82072265), Basic and applied basic research project jointly funded by Guangzhou University (College) (grant number: 202201020285 and 202201020276), Science and Technology Program of Guangzhou (grant number: 202102020074).

## Conflict of interest

The authors declare that the research was conducted in the absence of any commercial or financial relationships that could be construed as a potential conflict of interest.

## Publisher’s note

All claims expressed in this article are solely those of the authors and do not necessarily represent those of their affiliated organizations, or those of the publisher, the editors and the reviewers. Any product that may be evaluated in this article, or claim that may be made by its manufacturer, is not guaranteed or endorsed by the publisher.

## Figures and Tables

**Table:** 

3TC	lamivudine
AIDS	acquired immune deficiency syndrome
aOR	adjusted odds ratio
ART	antiretroviral therapy
AUROC	area under the receiver operator characteristic curve
CI	confidence interval
HBV	hepatitis B virus
HCV	hepatitis C virus
HIV	human immunodeficiency virus
IL-18	interleukin-18
IQR	interquartile range
LPV/r	lopinavir/ritonavir
ROC	receiver operating characteristic curve
TDF	tenofovir disoproxil fumarate
EFV	efavirenz
UNAIDS	Joint United Nations Program on HIV and AIDS.
