# Supplementary figures and images for: Early on-treatment plasma interleukin-18 as a promising indicator for long-term virological response in patients with HIV-1 infection
Source: Front Med (Lausanne). 2023 Jun 13;10:1170208. doi: 10.3389/fmed.2023.1170208 (PMC10294041; doi:10.3389/fmed.2023.1170208)

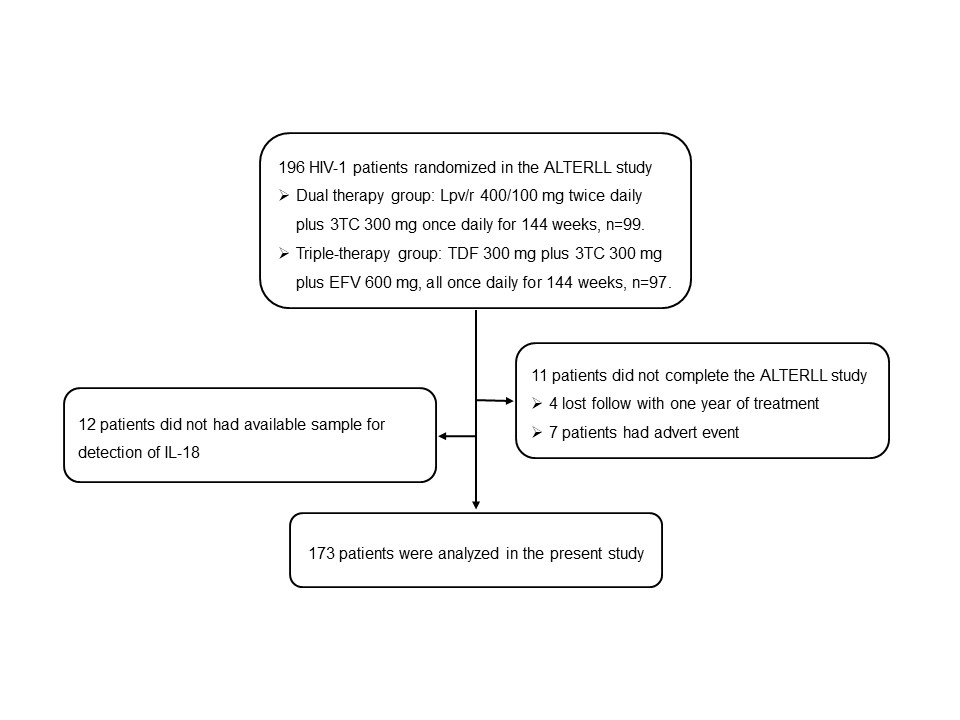

Supplement: Supplementary file 1 [file Image_1.JPEG]
