# Supplementary material for: Early on-treatment plasma interleukin-18 as a promising indicator for long-term virological response in patients with HIV-1 infection
Source: Front Med (Lausanne). 2023 Jun 13;10:1170208. doi: 10.3389/fmed.2023.1170208 (PMC10294041; doi:10.3389/fmed.2023.1170208)
Supplement: Supplementary file 2 [file Table_1.docx]

**Table S1: The primer sequences of PCR for** **amplification for sub-genotypes of the HIV-1 virus.**

| Primer name | Position in HXB2 | Primer sequence (5’-3’) |
| --- | --- | --- |
| Outer F1 | 2028-2050 | TTGGAAATGTGGAAAGGAAGGAC |
| Outer R1 | 3509-3539 | CTGTATTTCTGCTATTAAGTCTTTTGATGGG |
| Inner-F1 | 2147-2166 | CAGAGCCAACAGCCCCACCA |
| Inner-R1 | 3300-3326 | CTTCTGTATATCATTGACAGTCCAGCT |

**Table S2: Performance of Week 24 interleukin-18 level in predicting long-term virological response.**

| **Cut-off values**  **of IL-18** | **Sensitivity (%)** | **Specificity (%)** | **Yoden index** |
| --- | --- | --- | --- |
| 55 | 90.9 | 53.9 | 0.448 |
| 57 | 90.9 | 57.1 | 0.48 |
| 58 | 90.9 | 59.7 | 0.506 |
| 60 | 90.9 | 63.0 | 0.539 |
| 61 | 90.9 | 64.3 | 0.552 |
| 62 | 90.9 | 64.9 | 0.558 |
| 63 | 90.9 | 66.9 | 0.578 |
| 64 | 90.9 | 68.2 | 0.591 |
| 65 | 81.8 | 68.8 | 0.506 |
| 68 | 72.7 | 72.7 | 0.454 |

*Note*: The top ten Yoden index by sum of sensitivity and specificity of Week 24 IL-18 level are shown. Yoden index = Sensitivity - (1- Specificity).
